# The Mode of Action of Cyclic Monoterpenes (−)-Limonene and (+)-α-Pinene on Bacterial Cells

**DOI:** 10.3390/biom11060806

**Published:** 2021-05-29

**Authors:** Olga E. Melkina, Vladimir A. Plyuta, Inessa A. Khmel, Gennadii B. Zavilgelsky

**Affiliations:** 1 State Research Institute of Genetics and Selection of Industrial Microorganisms of the National Research Centre “Kurchatov Institute”, Kurchatov Genomic Center, 117545 Moscow, Russia; oligamelkina@gmail.com; 2 Institute of Molecular Genetics of the National Research Center “Kurchatov Institute”, 123182 Moscow, Russia; plyutaba@gmail.com (V.A.P.); khmel@img.ras.ru (I.A.K.)

**Keywords:** volatile organic compounds, (−)-limonene, (+)-α-pinene, luxbiosensors, stress, DnaKJE-refolding

## Abstract

A broad spectrum of volatile organic compounds’ (VOCs’) biological activities has attracted significant scientific interest, but their mechanisms of action remain little understood. The mechanism of action of two VOCs—the cyclic monoterpenes (−)-limonene and (+)-α-pinene—on bacteria was studied in this work. We used genetically engineered *Escherichia coli* bioluminescent strains harboring stress-responsive promoters (responsive to oxidative stress, DNA damage, SOS response, protein damage, heatshock, membrane damage) fused to the *luxCDABE* genes of *Photorhabdus luminescens.* We showed that (−)-limonene induces the P*katG* and P*soxS* promoters due to the formation of reactive oxygen species and, as a result, causes damage to DNA (SOSresponse), proteins (heat shock), and membrane (increases its permeability). The experimental data indicate that the action of (−)-limonene at high concentrations and prolonged incubation time makes degrading processes in cells irreversible. The effect of (+)-α-pinene is much weaker: it induces only heat shock in the bacteria. Moreover, we showed for the first time that (−)-limonene completely inhibits the DnaKJE–ClpB bichaperone-dependent refolding of heat-inactivated bacterial luciferase in both *E. coli* wild type and mutant Δ*ibpB* strains. (+)-α-Pinene partially inhibits refolding only in Δ*ibpB* mutant strain.

## 1. Introduction

In recent years essential oils have attracted significant scientific interest because they exhibit a broad spectrum of bioactivities, such as antibacterial, antifungal, antiviral, and insecticidal activities [1,2,3]. Moreover, their main active compounds—aldehydes, terpenes, and phenols—are widely used according to the recommendations of the US Food and Drug Administration as food additives, providing a significant reduction in the level of microbial contamination [1,2,4]. Volatile terpenes are derived from the terpene-building units dimethylallyl pyrophosphate and isopentenyl pyrophosphate. However, recent studies have revealed that terpenes are produced not only by plants but also by bacteria, fungi, and amoebae [5,6,7].

**Figure 1:**
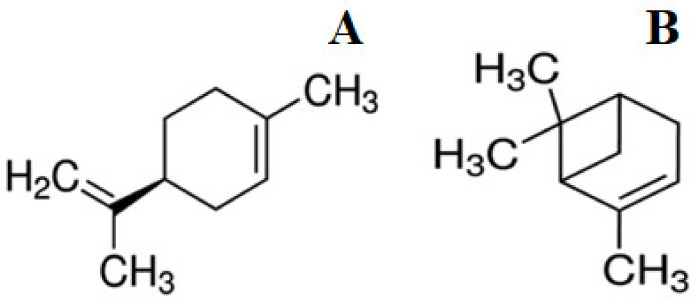
The chemical structure of (−)-limonene (**A**) and (+)-α-pinene (**B**).

Volatile organic compounds (VOCs) limonene and pinene are of great interest in terms of practical application. They are widespread in plants: lemon and other citrus fruits contain limonene, and the main active compound of essential oil obtained from conifers is pinene. These compounds belong to the class of VOCs known as cyclic monoterpenes (Figure 1). Limonene and α-pinene inhibit the growth of *Bacillus strains*, *Staphylococcus aureus*, *Listeria monocytogenes*, *Salmonella enterica*, *Saccharomyces cerevisiae*, *Zygosaccharomyces rouxii*, *Sclerotinia sclerotiorum*, and *Rhizoctonia solani* [6,7,8,9,10,11,12]. Some studies on the enantiomers of limonene and pinene have shown that the enantiomeric configuration influences biological activity [10,11,12]. For example, it was revealed that in general, (−)-limonene is more active than (+)-limonene [11], and only the positive enantiomers of pinene have antimicrobial activity against bacterial and fungal cells [10]. The studies mainly aimed to identify these VOCs’ biological activity [12], but their detailed mechanisms of action remain little understood. It was found that limonene, if present in the growth medium during incubation for 4–24 h, destroys the cell membrane of *Z. rouxii* yeast cells, which is accompanied by leakage of nucleic acids and proteins, as well as the degradation of proteins and their synthesis inhibition [8]. An increase in permeability and degradation of the cell membrane of Gram-negative *E. coli* and Gram-positive *S. aureus* bacteria were also shown when they were incubated in a nutrient medium containing finger citron essential oil (FCEO) with limonene (~50%) and also α-pinene as the main components [13]. Biological activities related to limonene and α-pinene demonstrate the importance of these terpenes and their optical isomers as promising therapeutic agents [3,14]. Limonene and α-pinene have been shown to have the potential as enhancers of antituberculosis drugs (ethambutol, rifampicin, and isoniazid) [5].

Luxbiosensors are *Escherichia coli* cells that contain a hybrid plasmid with two main inserted elements—a regulatory system (a promoter–operator region) and a group of transcriptionally fused reporter genes, *luxCDABE*. Luxbiosensors are widely used to investigate the functional and ecological role (environmental monitoring) of compounds with different chemical structures [15,16,17,18,19,20,21].

In this paper, we present the results on the reactions of microorganisms after the addition of (−)-limonene and (+)-α-pinene to the nutrient medium. These results are obtained using genetically engineered *Escherichia coli* strains harboring stress-responsive promoters fused to the bioluminescent reporter gene system of *lux* operon.

## 2. Materials and Methods

### 2.1. Bacterial Strains

*Escherichia coli* strains used in this study are listed in Table 1. The bacterial *E. coli* K12 strains MG1655, JW3914-1, JW3933-3, QC868, and QC871 were used to measure the activity of VOCs as inductors of oxidative stress, SOSresponse, and heatshock.

The *E. coli* strains BW25113 and JW3663 *ibpB::kan* were used to measure the activity of VOCs as inhibitors of the DnaKJE-dependent refolding.

The *E. coli* strains MG1655, QC871, JW3914-1, and JW3933-3 were used to measure the antibacterial activity of VOCs.

### 2.2. Plasmids

The plasmids pIbpA’::lux (for detection of heat shock), pColD’::lux (for detection of SOS response), pKatG’::lux, and pSoxS’::lux (for detection of oxidative stress) were constructed before [20]. The plasmid pFabA’::lux [17,19,21] was used for the detection of membrane damage. The plasmids pMerR’::lux, pCopA’::lux, and pZntA’::lux were used as the negative control [26,27]. Vector pDEW201(Ap^r^), containing the *Photorhabdus luminescensluxCDABE* genes without a promoter [28], was the parent plasmid for the genetic constructs.

The plasmid pXen7 containing the *P. luminescensluxCDABE* genes under the *lac* promoter was used [29] to measure the inhibition of bacterial luciferaseenzymatic activity by (−)-limonene and (+)-α-pinene.

Kinetics and the level of the DnaKJE-dependent refolding of heat-inactivated luciferase were measuredin vivoin *E. coli* cells containing pLeo1 plasmid. The plasmid pLeo1 is the pUC18 derivative bearing the *Photobacterium leiognathi luxCDABE* genes encoding the α and β subunits of luciferase and reductase under the control of the *lac* promoter [30].

The plasmid pLR was used to measure the membrane permeability for D-luciferin—substrate of firefly luciferase. The plasmid pLR (Ap^r^) (kindly provided by Dr. N.N. Ugarova, Moscow State University, Russia) contains the *Luciola mingrelicaluc* geneunder the *P_r_* promoter of *Aliivibrio fischeri lux* operon [31].

Constructed hybrid plasmids were introduced into the cells of various strains of *E. coli*. Excretion of plasmid DNA, restriction and ligation of DNA fragments, and transformation of cells were conducted according to [32].

### 2.3. Nutrient Media and Growth Conditions

Bacterial strains were cultivated in the liquid Luria–Bertani (LB) medium or on Petri dishes with agarized LB medium (LA) supplemented with appropriate antibiotics. The cells were grown under aeration at 30 °C until the early exponential phase. Antibiotics were added at the following concentrations: streptomycin (Sm; 25 µg/mL), kanamycin (Km; 20 µg/mL), ampicillin (Ap; 100 µg/mL), chloramphenicol (Cm; 25 µg/mL).

### 2.4. Enzymes and Chemicals

All the chemicals were of analytical grade. Antibiotics (streptomycin, kanamycin, chloramphenicol, and ampicillin) were obtained from Sigma Chemical Co (St. Louis, MO, USA), Merck (Darmstadt, Germany), and Biopharm (Moscow, Russia). Methyl viologen (paraquat), mitomycin C, dimethyl sulfoxide (DMSO), D-luciferin, and VOC compounds (−)-limonene (96% purity, CAS # 5989-54-8) and (+)-α-pinene (98% purity, CAS # 80-56-8) were purchased from Sigma-Aldrich Chimie GmbH (Steinheim, Germany). Triton X-100 was purchased from ServaElectrophoresis GmbH (Heidelberg, Germany), andethanol (96%) was purchased from LLC Donskoy (Tula region, Kimovsky district, rp. Epifan, Russia). Hydrogen peroxide (H_2_O_2_), HgCl_2_, and ZnSO_4_ were purchased from Merck (Darmstadt, Germany), CuSO_4_ was purchased from Sigma-Aldrich Chimie GmbH (Steinheim, Germany).

We used an uncoupler of oxidative phosphorylation protonophore carbonyl cyanide—3-chlorophenylhydrazone (CCCP)—to reduce the ATP content in cells. CCCP was obtained from Sigma-Aldrich Chimie GmbH (Steinheim, Germany). ATP concentration was assayed with firefly luciferase [33].

The required concentration of stock solutions of paraquat, H_2_O_2_, Triton X-100, and HgCl_2_was obtained by dissolving the compounds mentioned above in distilled water.

The enzymes used in this work were provided by Fermentas (Vilnius, Lithuania). According to manufacturer protocol, the cell lysate containing the firefly luciferase was obtained using the Dual-Luciferase Reporter Assay System (Promega, Madison, WI, USA).

All substances were stored at a temperature specified by the manufacturer. All test solutions were prepared just before their use.

### 2.5. Measurement of Production and Activity of Bacterial Luciferase

Bacterial luciferase (E) catalyzes the oxidation of long-chain aldehydes (RCHO) by atmospheric oxygen (O_2_) in the presence of reduced flavin mononucleotide (FMNH_2_):

FMNH_2_ + RCHO + O_2_→ FMN + RCOOH + H_2_O + a quantum of light (λ_max_ = 490 nm) (1)

Recombinant luminescent bacteria (luxbiosensors) were pregrown at 30 °C in LB medium supplemented with appropriate antibiotics overnight. Then the culture was diluted up to 10^7^ CFU/mL by fresh LB and grown at 30 °C under aeration until the early exponential phase. The cell aliquots (200 μL)were placed in special cuvettes, one serving as a control to which 4 µL of DMSO was added, while 4 µL of (−)-limonene or (+)-α-pinene in DMSO solution in various concentrations were added in the others. The prepared samples with lux-biosensor cells were placed in front of a photomultiplier in the Luminometer Photometer LMA01 (Beckman Coulter, Praha, Czech Republic). After selected time intervals, the intensity of the bioluminescence of the cellular suspension was measured. Luminescence values were expressed in terms of the instrument’s arbitrary relative light units (RLU). The samples were incubated at room temperature. Three main parameters that characterize the quality of the luxbiosensor were estimated: response amplitude (RA) or induction factor (R) (if the luminescence intensity of the control preparation is practically constant in the time interval 0–t), the minimal response time (t_min_), and threshold sensitivity (P)—the concentration of the inductor when RA or R is approximately equal to 2. The RA was determined by the formula RA = I_t_− I_0_/I_k_− I_0_, where I_0_ is the intensity of the preparation bioluminescence at the moment of inductor addition (t = 0), I_k_ is the intensity of the bioluminescence of the control preparation (in the absence of the inductor) at moment t, and I_t_ is the intensity of the bioluminescence of the test preparation at moment t. Induction factor R = I_t_/I_0_.

For induction of luxbiosensors with the P*katG*, P*soxS*, P*colD*, P*fabA*, and P*ibpA* promoters, various concentrations of H_2_O_2_, paraquat (stimulate the formation of superoxide anion radical (O_2_^.−^)), mitomycin C (forms both DNA adducts and interstrand cross-links [34]), Triton X-100, and ethanol, respectively, were used as a positive control.

### 2.6. Thermal Inactivation and Refolding

(−)-Limonene and (+)-α-pinene were placed in small plastic tubes (volume 1 mL) containing bacterial cells (2–3 × 10^8^ CFU/mL) in LB medium. The tubes were tightly sealed with two layers of Parafilm M (Pechiney Plastic Packaging Company, Chicago, IL, USA). The controls were performed in the absence of terpenes. For in vivo heatinactivation of *Photobacterium leiognathi* luciferase, bacterial cells were transferred to 46 °C in a water bath for 5 min. The production of luciferase and heat shock proteins was stopped by adding chloramphenicol to a final concentration of 167 μg/mL. For subsequent recovery (refolding) of *P. leiognathi* luciferase, bacterial cells were transferred back to lower temperatures (22 °C). The cell aliquots (200 μL) were transferred to a luminometer in which bioluminescence intensity was measured as a function of the incubation time, and the values were plotted as a percentage of the initial activity. For the highest DnaKJE and ClpB levels, *E. coli* cells were first incubated at 42 °C for 30 min without chloramphenicol (preliminary “heat shock”). All experiments were repeated four to six times with two to three tubes per experiment.

### 2.7. Antibacterial Activity

Antibacterial activity of (−)-limonene and (+)-α-pinene was tested using the agar diffusion method [35]. First, 100 µL of bacterial suspension (10^7^ CFU/mL) was spread on nutrient agar (NA) medium. Then, a sterile filter paper disc (diameter = 6 mm) containing 4 µL (3.36 mg) of the (sample) (−)-limonene was placed on the surface of the plate. All the plates incubating at 37 °C for 18 h were observed for zones of growth inhibition. The inhibition zones were measured in millimeters from the circumference of the discs to the circumference of the inhibition zone. All the assays were carried out in triplicate.

## 3. Results

### 3.1. The Action of (−)-Limonene and (+)-α-Pinene on the LuxBiosensors

#### 3.1.1. Oxidative Stress

**Figure 2:**
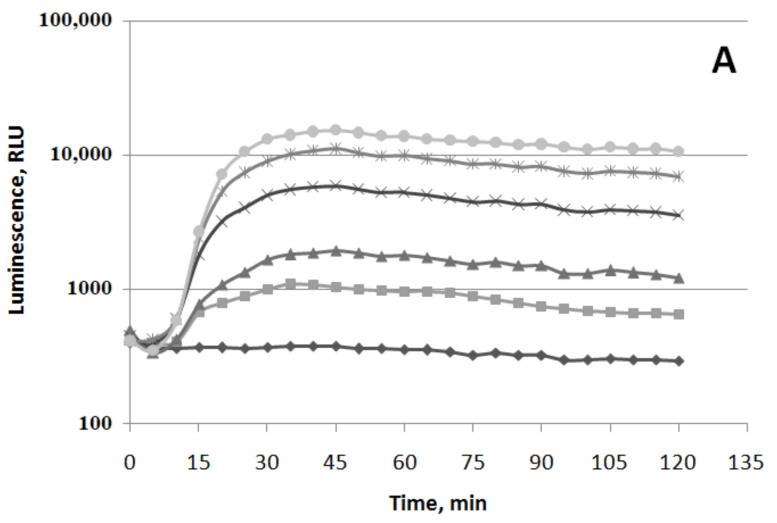

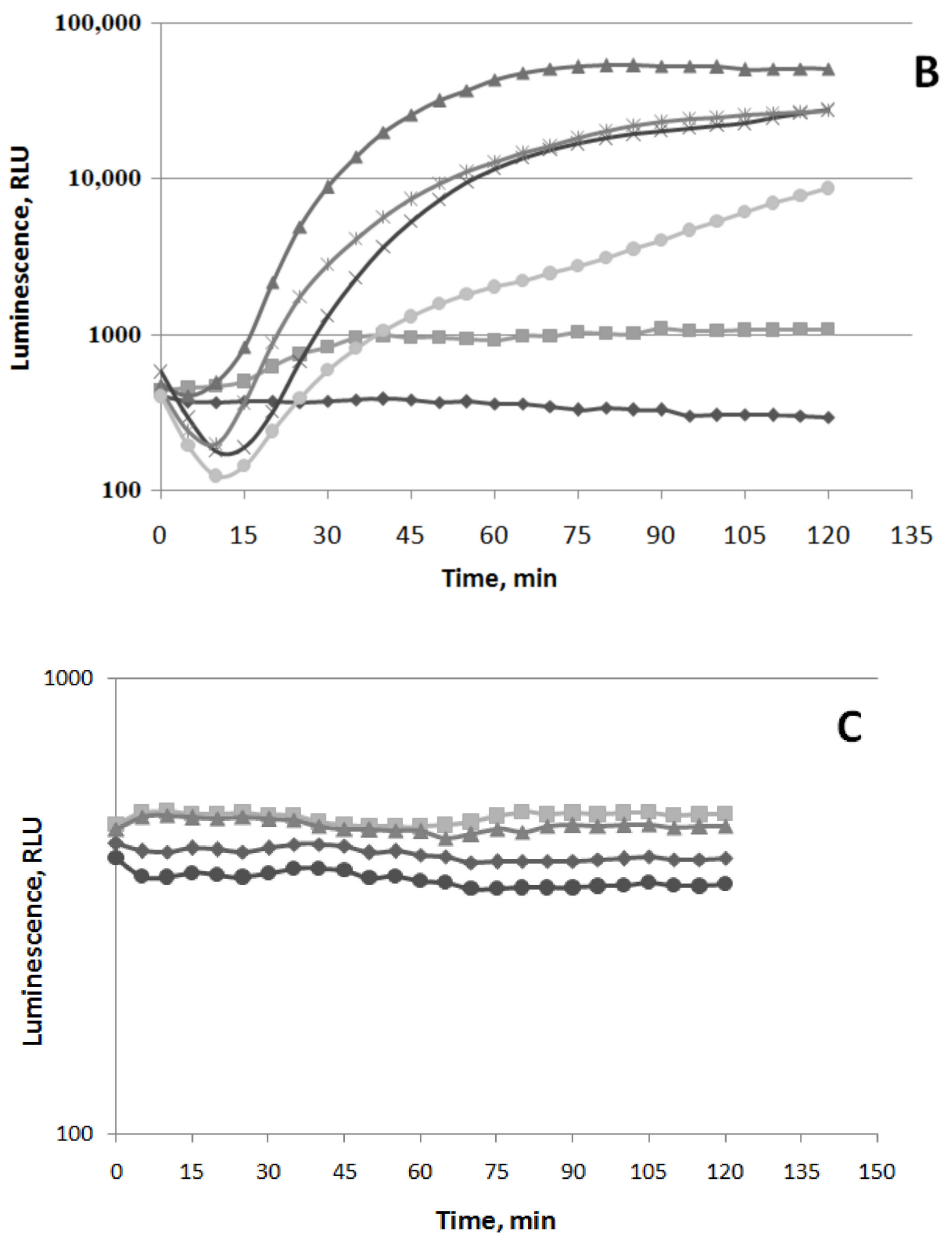
Luminescent response of strain *E. coli* MG1655 (pKatG’::lux) to hydrogen peroxide (**A**), (−)-limonene (**B**), and (+)-α-pinene (**C**). Time course of mean values of light emission (in RLU) in the presence of different chemical concentrations (**A**,**B**): (◆) 0 μM (black diamond—Control), (◼) 1 μM (gray square), (▲) 5 μM (dark triangle), (×) 25 μM (black cross), (🞶) 50 μM (gray asterisk), and (⬤) 100 μM (light circle); (**C**): (◆) 0 μM (black diamond—control), (◼) 5 μM (light square), (▲) 50 μM (gray triangle), (⬤) 100 μM (black circle).

**Figure 3:**
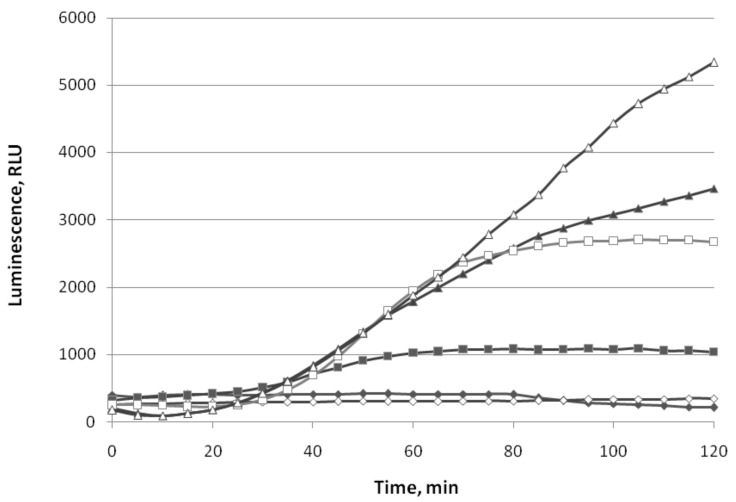
Effect of Δ*katG* mutation on the induction of the P*katG* promoter by (−)-limonene. Luminescent response of wild-type strain (*E. coli* MG1655 (pKatG’::lux)—dark symbols) and Δ*katG* mutant strain (*E. coli* JW3914-1 Δ*katG729::kan* (pKatG’::lux)—light symbols) to (−)-limonene. Kinetics of changes in mean values of light emission (in RLU) at various concentrations of (−)-limonene: (◆, ◇) 0 μM (control), (◼, ▢) 1 μM, (▲, △) 10 μM.

**Figure 4:**
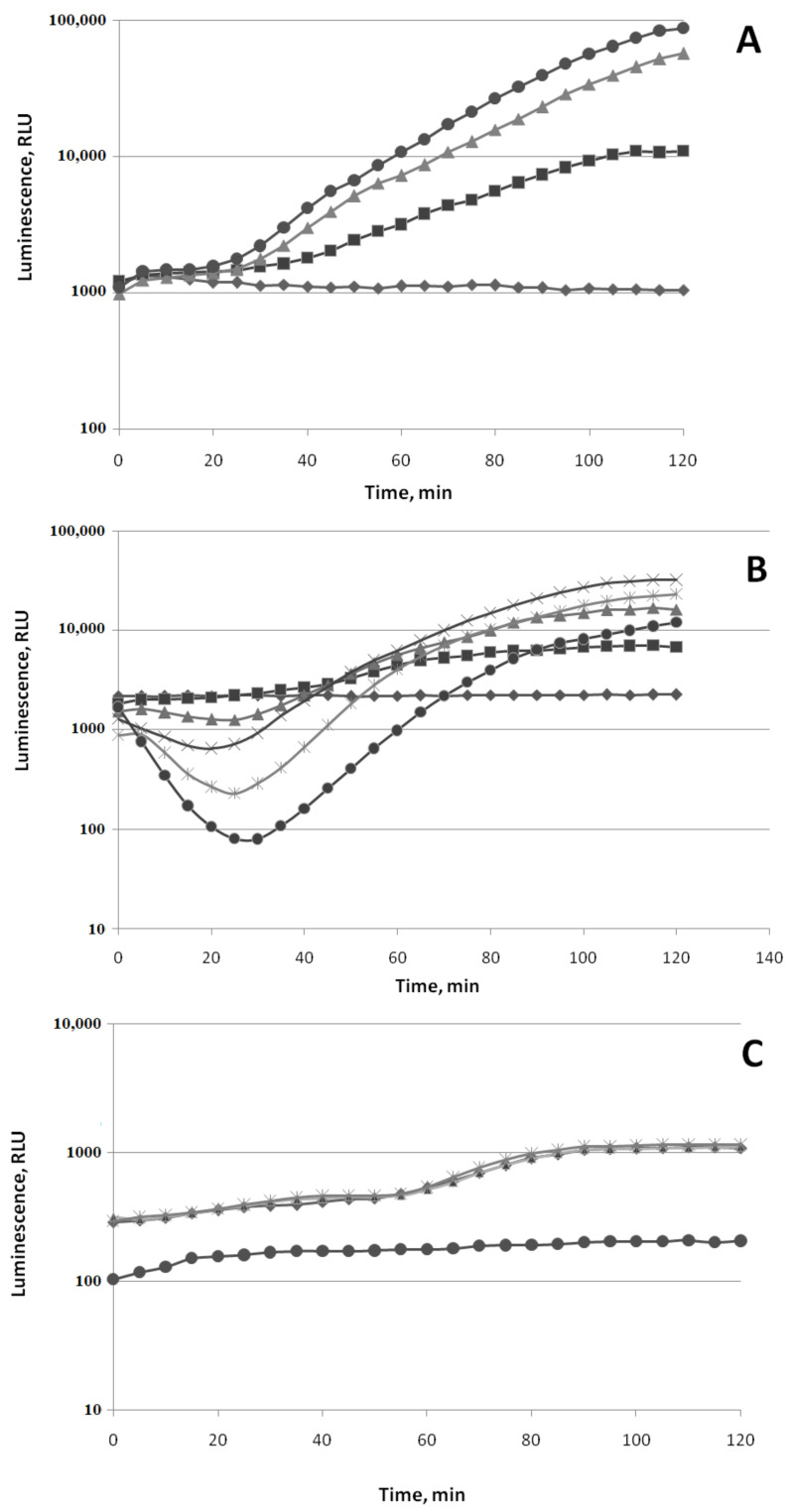
Luminescence response of strain *E. coli* MG1655 (pSoxS’::lux) to paraquat (methyl viologen) (**A**), (−)-limonene (**B**), and (+)-α-pinene (**C**). Kinetics of changes in mean values of light emission (in RLU) at various chemical concentrations: (**A**): (◆) 0 μM (control), (◼) 5 μM, (▲)—50 μM, (⬤) 100 μM; (**B**): (◆) 0 μM (control), (◼) 1 μM, (▲) 5 μM, (**×**) 25 μM, (🞶) 50 μM, (⬤) 100 μM; (**C**): (◆) 0 μM (control), (▲) 5 μM, (🞶) 50 μM, (⬤) 100 μM.

The induction of oxidative stress by VOCs, hydrogen peroxide, and paraquat is shown in Figure 2, Figure 3 and Figure 4. The experiment was carried out with the luxbiosensors responsive to oxidative stress—*E. coli* MG1655 (pKatG’::lux) and MG1655 (pSoxS’::lux) strains. Different concentrations of H_2_O_2_ increased the bioluminescence intensity of *E. coli* MG1655 (pKatG’::lux) (Figure 2A).

**Figure 5:**
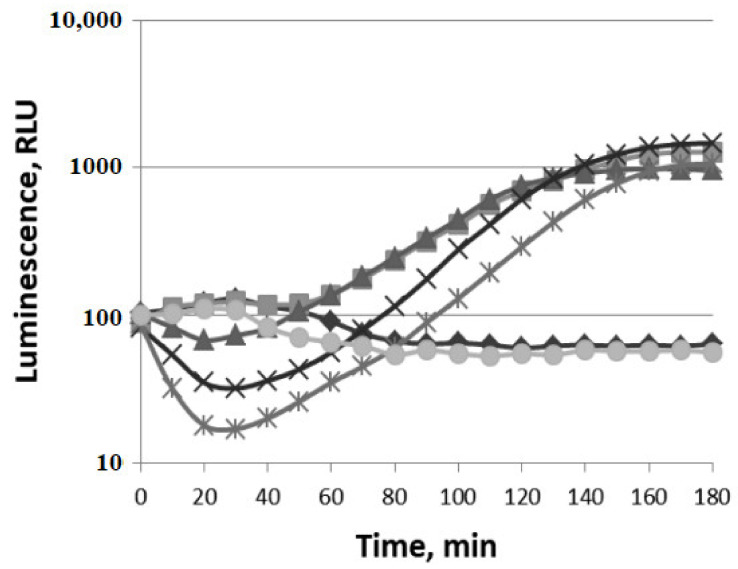
Luminescence response of strain *E. coli* MG1655 (pColD’::lux) to mitomycin C, (−)-limonene, and (+)-α-pinene. Kinetics of changes in mean values of light emission (in RLU) at various chemical concentrations: (◆) 0 μM (control); (◼) mitomycin C 10 μM; (⬤) (+)-α-pinene 100 μM; (−)-limonene (▲) 5 μM, (×) 25 μM, and (🞶) 100 μM.

**Figure 6:**
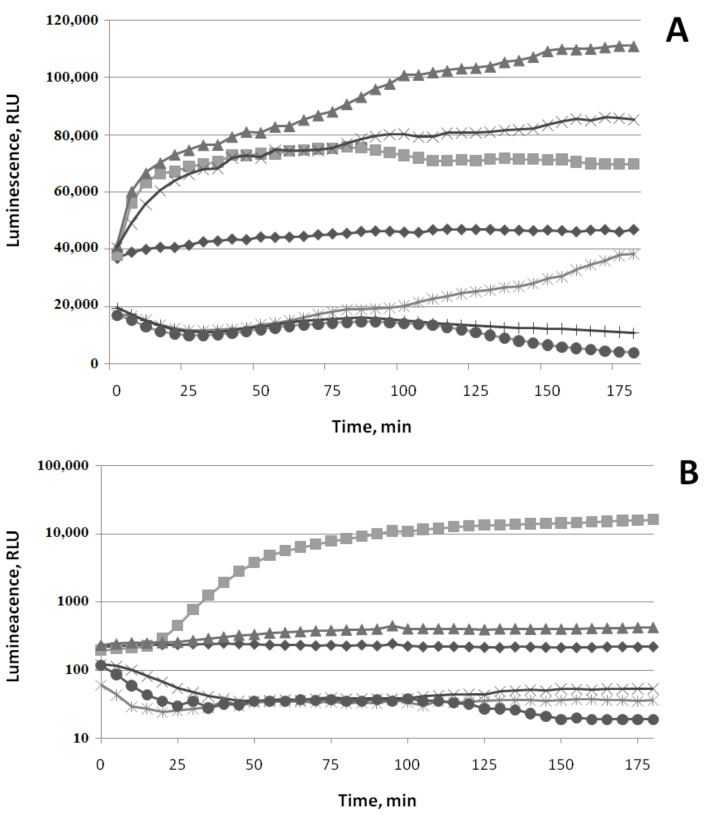
Luminescent responses of strains *E. coli* MG1655 (pFabA’::lux) to Triton X-100 and (−)-limonene (**A**) and *E. coli* MG1655 (pMerR’::lux) to HgCl_2_ and (−)-limonene (**B**). Kinetics of changes in mean values of light emission (in RLU) at various chemical concentrations: (**A**): (◆) Control (0 μM); TritonX-100 (◼) 0.5 μg/mL, (▲) 2 μg/mL, and (×) 4 μg/mL; (−)-limonene (🞶) 2 μM, (+) 4 μM, and (⬤) 10 μM. (**B**): (◆) Control (0 μM); HgCl_2_(▲) 0.001 μM and (◼) 0.3 μM; (−)-limonene (×) 2 μM, (🞶) 4 μM, and (⬤) 10 μM.

**Figure 7:**
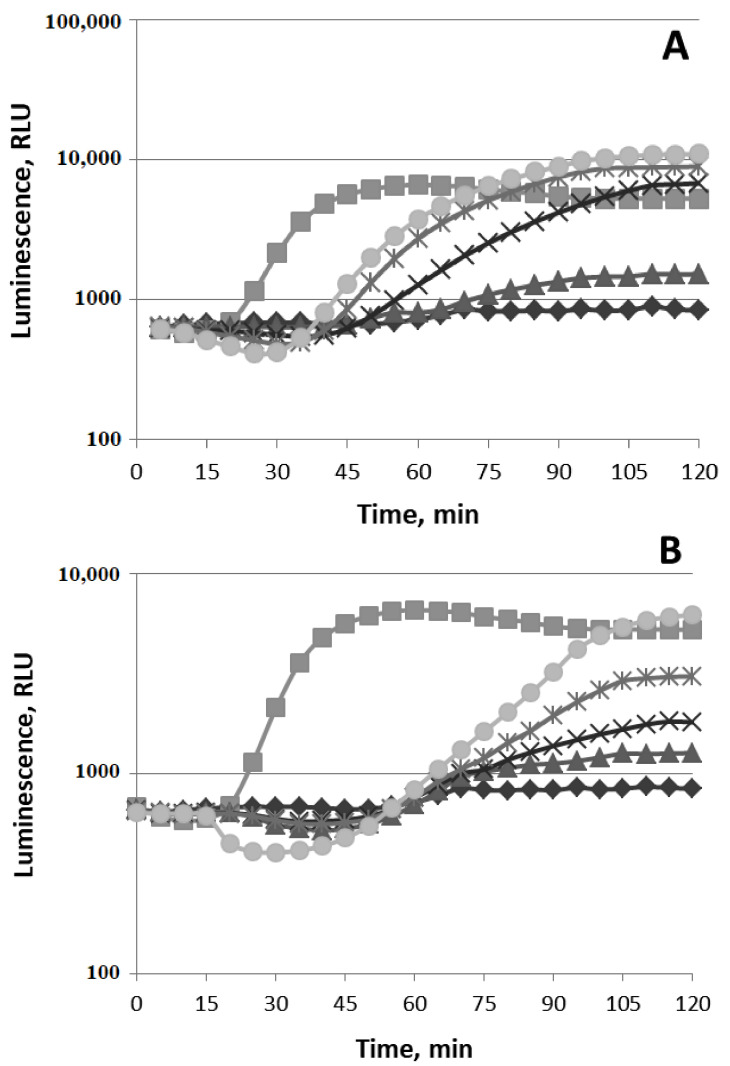
Luminescence response of *E. coli* MG1655 (pIbpA’::lux) (“heatshock”) to(−)-limonene (**A**) and (+)-α-pinene (**B**). Kinetics of changes in mean values of light emission (in RLU) at various chemical concentrations: (**A**,**B**): (◆) 0 μM (control); (◼) ethanol (4%); (▲) 5 μM, (×) 10 μM, (🞶) 15 μM, and (⬤) 20 μM of (−)-limonene (**A**) and (+)-α-pinene (**B**).

Data shown in Figure 2, Figure 3, Figure 4, Figure 5, Figure 6 and Figure 7 are mean values of light emission units (RLU) in at least four experiments. Standard deviations from the mean in these experiments did not exceed 25%.

The induction factor (R) gradually increases up to 6–7 times at 5 μM of H_2_O_2_, up to 15–20 times at 25 μM, up to 40 times at 50 μM, and up to 60 times at a concentration of 100 μM. The minimal incubation time required for inducing luminescence (t_min_) is ~10 min. It should be noticed that the maximum of induction factor R for all concentrations is reached at ~35–40 min, and then it almost does not decrease. The action of different concentrations of (−)-limonene (1–100 μM) is shown in Figure 2B. (−)-Limonene at concentration 1 μM (P—threshold sensitivity) increases bioluminescence by 2–3 times. Initially, a slight decrease in bioluminescence by 1.5 times at concentration 5 μM and by 5–6 times at 100 μM is observed. Then, with the increase in incubation time, the luminescence rapidly increases at 5 μM up to 80 times compared to the control (which is comparable to the action of ~50–100 μM of H_2_O_2_, Figure 2A) and after 50 min reaches a plateau. The induction factor R decreases at higher concentrations of (−)-limonene, e.g., at 100 μM, R = 15. Different concentrations of (+)-α-pinene (up to 100 μM) do not induce the P*katG* promoter (Figure 2C).

The curves of induction of the P*katG* promoter by (−)-limonene in the wild-type strain (*E. coli* MG1655 (pKatG’::lux)) and the Δ*katG* mutant strain (*E. coli* JW3914-1 Δ*katG* (pKatG’::lux)) are shown in Figure 3.

There is a significant increase in the induction factor R in the Δ*katG* mutant strain compared to the wild-type strain. (−)-Limonene at concentration 1 μM causes increased luminescence intensity by ~3 times in the wild-type strain and 10–15 times in the Δ*katG* mutant. The higher concentration of (−)-limonene (10 μM) causes the same patterns. A high level of induction of the P*kat*G promoter under the action of (−)-limonene, comparable to the effect of hydrogen peroxide, is observed. Thus, the absence of catalase enzyme in the bacterial cell in strain JW3914-1 Δ*katG729::kan* leads to the significant increase in the induction factor R in the Δ*katG* mutant strain compared to wild-type strain and indicates that the mechanism of action of (−)-limonene is associated with the formation of hydrogen peroxide in this process.

The data on the action of different concentrations of (−)-limonene and (+)-α-pinene on the P*soxS* promoter are represented in Figure 4.

Different concentrations of paraquat (methyl viologen) increase bioluminescence intensity of *E. coli* MG1655 (pSoxS’::lux) (Figure 4A). The induction factor R gradually increases over2 h: up to 10 times at a concentration of 5 μM of paraquat, up to 80–90 timesat 50 μM, and up to 100 times at 100 μM. The minimal incubation time required for inducing luminescence (t_min_) is ~20–25 min. The action of different concentrations of (−)-limonene is shown in Figure 4B. The minimum used concentration of (−)-limonene (1 μM) is the same as in the case of induction of the P*katG* promoter. However, the induction factor R of *E. coli* MG1655 (pSoxS’::lux) is lower compared to the R of *E. coli* MG1655 (pKatG’::lux): R does not exceed 10 times at 5 and 10 μM of (−)-limonene, and at 100 μM, at first, the luminescence decreases by about an order of magnitude and then starts to increase, crosses the control line (luminescence values of strain without VOCs) after 70 min, and after 2 h of incubation exceeds the control by ~5 times. The action of different concentrations of (+)-α-pinene does not induce the P*soxS* promoter. However, at a high concentration (100 μM), it reduces the intensity of bacterial luminescence by ~2–3 times (Figure 4C).

Summarizing the obtained data on the induction of oxidative stress by VOCs, we can conclude that in the bacterial cells, (−)-limonene promotes the synthesis of a significant amount of reactive oxygen species (ROS)—hydrogen peroxide and superoxide anion radical. (+)-α-Pinene is not active in this respect.

#### 3.1.2. SOSResponse

The data on the action of different concentrations of (−)-limonene and (+)-α-pinene on the SOSpromoter (P*colD*—DNA damage) of the plasmid pColD are presented in Figure 5.

Mitomycin C at a concentration of 10 μM increases bioluminescence intensity of *E. coli* MG1655 (pColD’::lux): the induction factor R gradually increases up to 10 times, with a t_min_of ~40 min (Figure 5). (−)-Limonene induces the P*colD* promoter, starting from a concentration of 5 μM, and, like mitomycin C, after ~40 min of incubation. At higher concentrations (25–100 μM), at first, the luminescence intensity decreases within 20–30 min of incubation up to 5–6 times and then increases and reaches a plateau after 2 h. The induction factor Rrises no more than 20 times at concentrations of (−)-limonene ≤ 100 μM. (+)-α-Pinene does not induce the P*colD* promoter at concentrations ≤ 100 μM.

#### 3.1.3. Cell Membrane Damage

The data on the action of different concentrations of (−)-limonene (2, 4, and 10 µM) on the P*fabA* promoter (cell membrane damage) are represented in Figure 6A.

Different concentrations of Triton X-100—0.5, 2, and 4.0 µg/mL(used as a positive control [17])—increase bioluminescence intensity of *E. coli* MG1655 (pFabA’::lux). The response amplitude RA gradually increases up to 3–4 times at a concentration of 2 µg/mLafter 2.5 h of incubation (Figure 6A). (−)-Limonene significantly decreases the luminescence intensity of *E. coli* MG1655 (pFabA’::lux) in the first minutes of incubation, but then after ~60–70 min of incubation at low concentrations (less than 5 µM), the luminescence intensity begins to gradually recover up to the control level (luminescence of strain without VOCs). At higher concentrations of (−)-limonene, inhibition of luciferase enzymatic activity does not allow evaluating the ability of this VOC to activate the P*fabA* promoter (Figure 6A).

(−)-Limonene does not induce the activity of the P*merR* (Figure 6B), P*copA,* and P*zntA* promoters (data not shown), which specifically react exclusively to mercury, copper, and zinc ions, respectively (*E. coli* MG1655 (pMerR’::lux), MG1655 (pCopA’::lux) and MG1655 (pZntA’::lux) are used as a negative control [26,27]).

#### 3.1.4. HeatShock

The data on the action of different concentrations of (−)-limonene and (+)-α-pinene on the P*ibpA* promoter (protein damage, heat shock) are represented in Figure 7.

Ethanol (4%) (used as a positive control [15])increases the bioluminescence intensity of *E. coli* MG1655 (pIbpA’::lux). The response amplitude RA gradually increases up to 10 times and reaches a plateau after ~40–45 min of incubation, with a t_min_of ~25 min (Figure 7A). The action of (−)-limonene leads to the induction of heat shock (Figure 7A). At the minimum concentration (P = 5 μM) of (−)-limonene, the luminescence begins to increase after ~60 min of incubation and then gradually increases by only 1.5–2 times. At 10 μM, the luminescence gradually increases by 8–10 times; at 15–20 μM, the luminescence begins to increase after 45 min of incubation and then increases up to 20 times after 90–100 min of incubation (Figure 7A).

(+)-α-Pinene induces the P*ibpA* promoter. The induction begins to increase after ~60 min of incubation; C_min_ = 5 μM. At 10 μM, the luminescence increases by 3 times; at 20 μM, the luminescence increases by up to 10 times (Figure 7B). However, the action of (+)-α-pinene on the induction of bioluminescence intensity of *E. coli* MG1655 (pIbpA’::lux) is weaker compared to the action of (−)-limonene.

#### 3.1.5. Effect of VOCs on the Enzymatic Activity of Native Bacterial Luciferase *P. luminescens*

**Figure 8:**
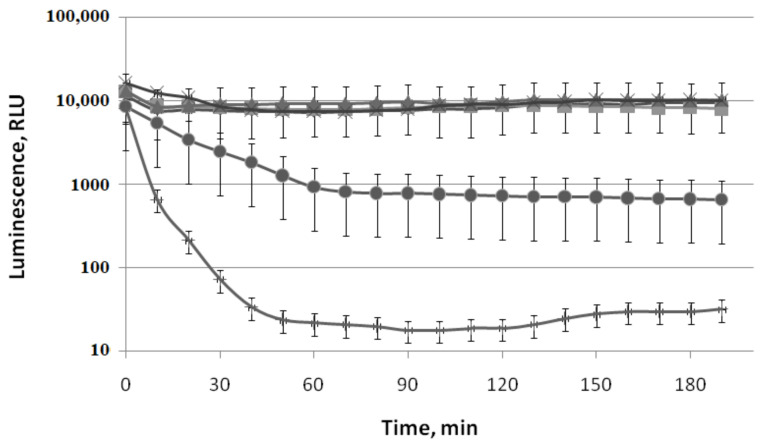
Effect of (−)-limonene and (+)-α-pinene on luciferaseenzymatic activity of *P. luminescens* in the *E. coli* MG1655 (pXen7) strain. Kinetics of changes in mean values of light emission (in RLU) at various chemical concentrations: (◆) 0 μM (control); (+)-α-pinene (◼) 1 μM, (▲) 10 μM, and (+) 100 μM; (−)-limonene (🞶) 1 μM, (⬤) 10 μM, and (×) 100 μM. The *ordinate axis* shows the enzymatic activity of luciferase. The *abscissa axis* shows the time of incubation at 22 °C.

The decrease in the intensity of cell luminescence is observed in the initial moment after adding (−)-limonene, and to a lesser extent (+)-α-pinene, to luxbiosensors (Figure 3, Figure 4B, Figure 5A, Figure 6 and Figure 7). In this connection, the action of (−)-limonene and (+)-α-pinene on luciferase enzymatic activity, contained in the bacterial cell, was studied. For this purpose, the *E. coli* MG1655 (pXen7) strain was used. The plasmid pXen7 contains *P. luminescens luxCDABE* genes under *lac* promoter that provide constitutive expression of *lux* genes [29]. The initial luminescence level of the cell suspension in the exponential phase is about 10,000 RLU (Figure 8).

After (−)-limonene is added into the cell suspension at a concentration of less than 5 μM, the luminescence intensity of *E. coli* MG1655 (pXen7) strain almost does not decrease during subsequent incubation at room temperature. However, a significant decrease in luminescence intensity is observed at concentrations of 10 μM of (−)-limonene and especially at 100 μM, by 10 and 100 times, respectively (Figure 8). Furthermore, simple dilution or centrifugation of the bacterial suspension resulted in the recovery of 80–100% of the initial luminescence intensity (within experimental error). These data indicate that the inhibition effect seen at high concentrations of (−)-limonene was fully reversible. Consequently, the inhibition of luciferase enzymatic activity by (−)-limonene can be explained by the competition of these VOCs with the luciferase substrate, long-chain aldehyde, or FMNH_2_.

(+)-α-Pinene at concentrations up to 100 μM does not decrease the intensity of cell luminescence (Figure 8). It can be assumed that the insignificant inhibitory effect of (+)-α-pinene is determined by low solubility (2.49 mg/L) and weak penetration into the cell cytoplasm due to the connection of (+)-α-pinene with the membrane. This assumption is supported by the data obtained using cell lysate containing the firefly *L. mingrelica* luciferase (see Section 3.3).

### 3.2. Effect of (−)-Limonene and (+)-α-Pinene on the DnaKJE-Dependent Refolding of Heat-Inactivated Bacterial Luciferase

It was shown that such VOCs as ketones 2-heptanone, 2-nonanone, and 2-undecanone effectively inhibit the DnaKJE-dependent refolding of heat-inactivated bacterial luciferases only in the *E. coli* Δ*ibpB* mutant strain lacking small chaperone IbpB [36]. Chaperone IbpB, which forms a complex with hydrophobic sites in proteins [37,38,39], appears to inhibit the complexation of these sites with hydrophobic ketones. Therefore, it was of interest to determine the ability of terpenes (−)-limonene and (+)-α-pinene to inhibit the DnaKJE-dependent refolding of proteins depending on the presence of small chaperone IbpB in the cell. Kinetics and the level of the DnaKJE-dependent refolding of heat-inactivated luciferase were measuredin vivoin the wild-type strain(*E. coli* BW25113) and the Δ*ibpB* mutant strain (*E. coli* JW3663 *ibpB::kan*), containing plasmid pLeo1 with *P. leiognathilux CDABE* genes under control of the *lac* promoter, that provide constitutive expression of thesegenes.

**Figure 9:**
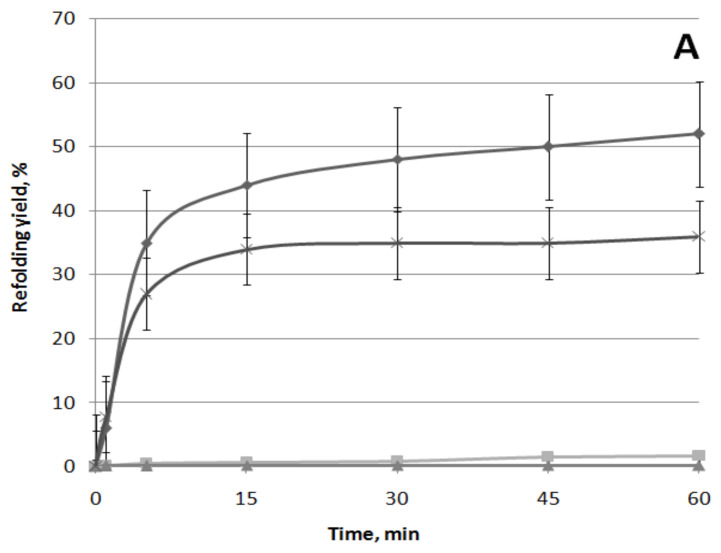

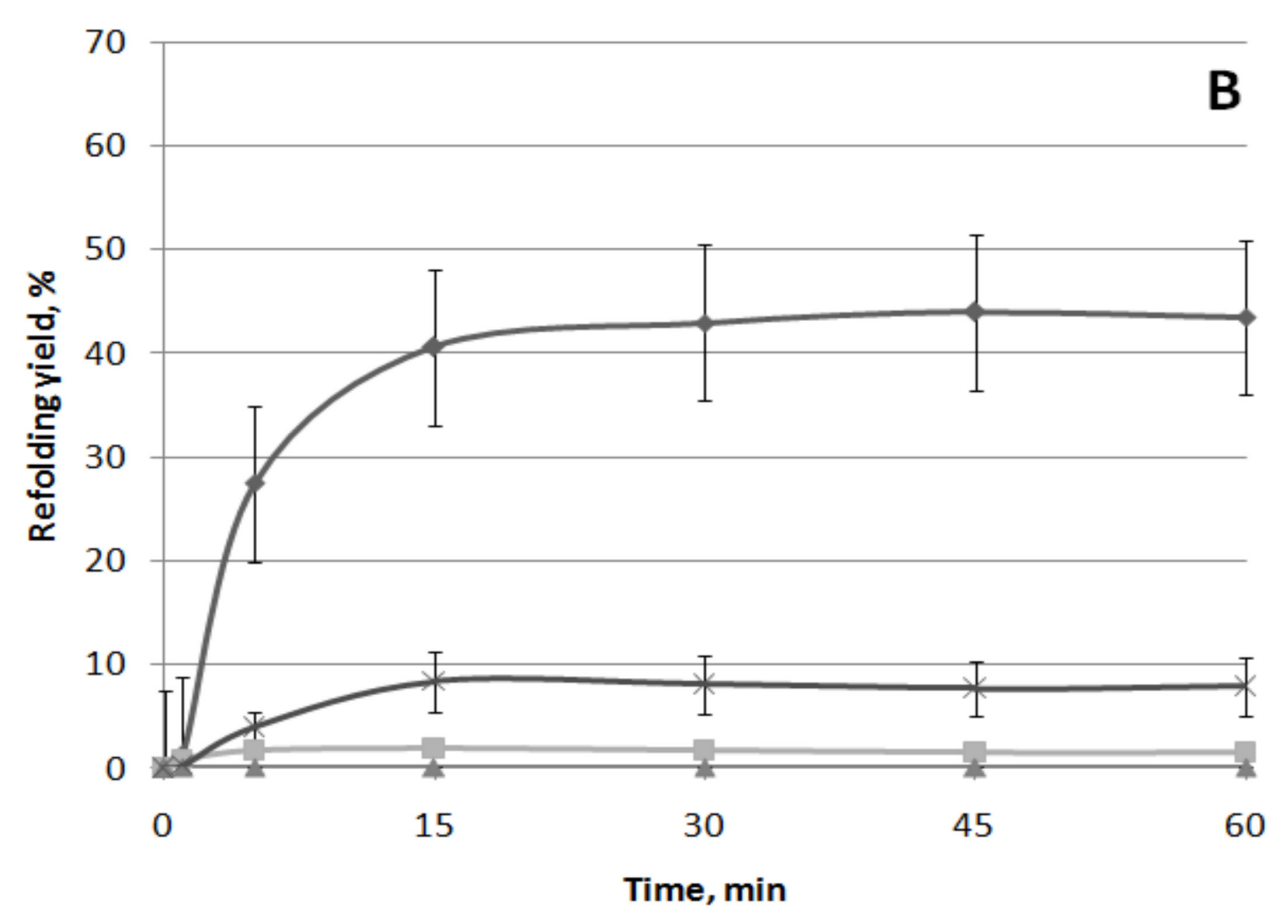
Effect of (−)-limonene, (+)-α-pinene, and CCCP on the kinetics of refolding of heat-inactivated *P. leiognathi* luciferase in the wild-type strain (*E. coli* BW25113 (pLeo1)) (**A**) and Δ*ibpB* mutant strain(*E. coli* JW3663 *ibpB::kan* (pLeo1)) (**B**). (◆) Control (0 μM), (×) 20 μM of (+)-α-pinene, (◼) 10 μM of (−)-limonene, (▲) 50 μM of CCCP. The *ordinate axis* shows the enzymatic activity of luciferase in the percentage of the initial level. The *abscissa axis* shows the time of incubation at 22 °C.

The effects of (−)-limonene, (+)-α-pinene, and CCCP on refolding kinetics of the heat-inactivated *P. leiognathi* luciferase in the wild-type strain (*E. coli* BW25113 (pLeo1)) and the Δ*ibpB* mutant strain (*E. coli* JW3663 *ibpB::kan* (pLeo1)) are shown in Figure 9.

Protonophore CCCP (3-chlorophenylhydrazone) was used as a positive control. The presence of protonophore CCCP (50 µM) in the medium leads to a decrease in the intracellular concentration of ATP to almost a minimum during the first few minutes and at the same time completely inhibits DnaKJE-dependent refolding of heat-inactivated luciferase both in the wild-type strain (Figure 9A) and the Δ*ibpB*mutant (Figure 9B). The action of (−)-limonene (10 µM) causes the same patterns (Figure 9). (+)-α-Pinene (20 µM), like ketones, only partially inhibits refolding in the Δ*ibpB* mutant strain (Figure 9B). However, (−)-limonene (10 µM) shows a significantly higher ability to compete with the small chaperone IbpB for binding to hydrophobic sites in the denatured macromolecule and the DnaKJE chaperone since it completely inhibits refolding not only in the Δ*ibpB* mutant strain but also in the wild strain BW25113 (Figure 9A).

### 3.3. Effect of (−)-Limonene and (+)-α-Pineneon Firefly Luciferase Activity

**Figure 10:**
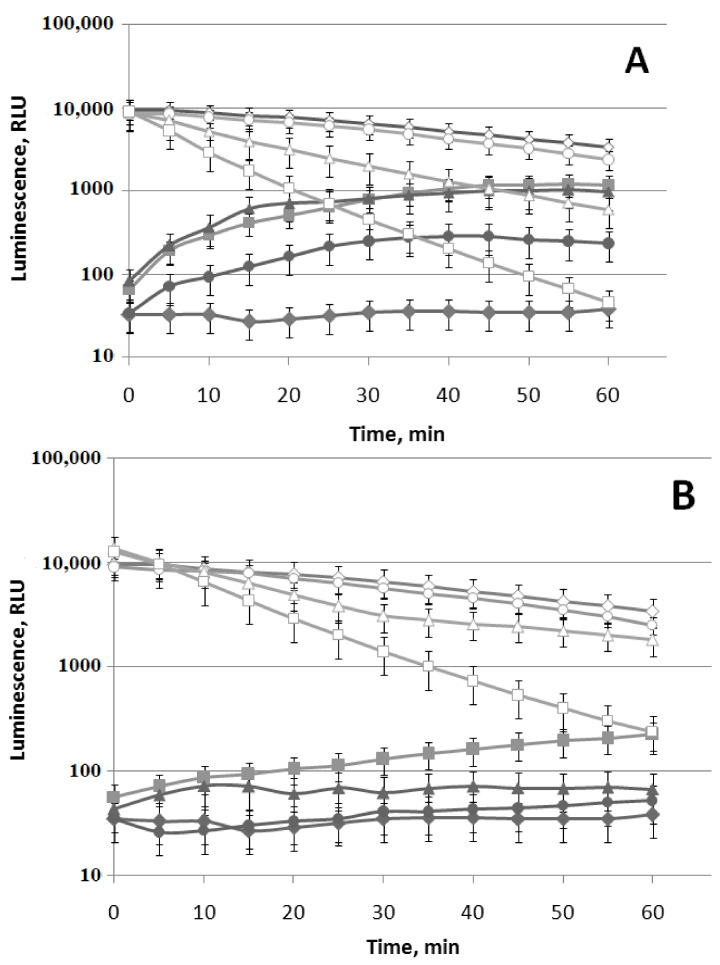
Effect of (−)-limonene (**A**) and (+)-α-pinene (**B**) on the activity of firefly *Luciola mingrelica* luciferase in *E. coli* MG1655 (pLR)living and lysed cells. Kinetics of changes in mean values of light emission (in RLU) at various chemical concentrations. (**A**,**B**): *E. coli* MG1655 (pLR) (dark symbol) and bacterial cell lysate (light symbol); (◆, ◇) 0 μM (control), (⬤,🞅) 1 μM, (▲,△) 10 μM, (◼,□) 100 μM. The *ordinate axis* shows the enzymatic activity of luciferase. The *abscissa axis* shows the time of incubation at 20 °C.

(−)-Limonene inhibits DnaKJE-dependent refolding, regardless of the presence of small chaperone IbpB in the cell, as entirely as the protonophore CCCP. Therefore it could be assumed that (−)-limonene, like the protonophore CCCP, decreases the intracellular concentration of ATP (ATP is a necessary factor for refolding with the ATP-dependent chaperones). The firefly *L. mingrelica* luciferase, whose enzymatic activity is utterly dependent on the presence of ATP, was used to test this hypothesis: ATP + O_2_+ D-luciferin → AMP + PP_i_+ CO_2_ + oxyluciferin + light (λ_max_ = 566 nm). The data on the action of different concentrations of (−)-limonene and (+)-α-pinene on the firefly luciferase luminescence intensity in *E. coli* MG1655 (pLR) strain and cell lysate are represented in Figure 10. The plasmid pLR contains *luc* gene encoding the *L. mingrelica* luciferase under the *P_r_* promoter of *A. fischeri lux* operon. The luminescence intensity gradually increased at all concentrations of (−)-limonene: the response amplitude (RA) at a concentration of 10 μM increases up to 5 and ~30 times after 10 and 30 min of incubation, respectively (Figure 10A). In comparison, the presence of protonophore CCCP (50 µM; used as a positive control) in the medium leads to a sharp decrease in luminescence intensity in *E. coli* MG1655 (pLR) (up to ~50–100 times from the initial level), which directly indicates a reduction in the intracellular concentration of ATP (data not shown). Therefore, the data show the absence of influence of (−)-limonene on the intracellular content of ATP in the early stages of action (in a period from 0 to 60 min of incubation).

The increase in the luminescence intensity of cells after adding (−)-limonene and (+)-α-pinene requires explaining this phenomenon. We assume that (−)-limonene and (+)-α-pinene, acting on cell membranes, increase their permeability for D-luciferin—a substrate of the luciferase, which very weakly penetrates bacterial membranes at pH 7.5 [40,41,42]. Confirmation of this assumption was obtained by adding (−)-limonene and (+)-α-pinene to firefly luciferase in anin vitroexperiment with *E. coli* MG1655 (pLR) living and lysed cells (Figure 10).

(−)-Limonene induces a gradual bioluminescence increase in *E. coli* MG1655 (pLR) cells without reducing the luminescence intensity of the cell lysate already at a concentration of 1 μM. However, at the concentration of 10 μM and especially at 100 μM, significant inhibition of luciferase enzymatic activity in the cell lysate is observed (Figure 10A). Different concentrations of (+)-α-pinene as well as (−)-limonene increase luminescence intensity in *E. coli* MG1655 (pLR) and, at the same time, decrease the luminescence intensity of cell lysates (Figure 10B). However, the activity of (+)-α-pinene is significantly lower than that of (−)-limonene.

### 3.4. Effect of (−)-Limonene and (+)-α-Pinene on the Growth of Bacteria

Antibacterial activity of (−)-limonene and (+)-α-pinene was tested using the agar diffusion method [13]. DIZs (diameters of inhibition zone) of different *E. coli* strains for (−)-limonene (in an amount of 3.36 mg (4 µL per sterile filter paper disc))are presented in Table 2. In this experiment, *E. coli* strains JW3914-1 Δ*katG729::kan* and JW3933-3 Δ*oxyR749::kan* were used to determine the involvement of antioxidant enzymes in cell resistance to the action of VOCs. The obtained data (Table 2) demonstrate that the inhibitory effect of (−)-limonene is significantly enhanced in the absence of catalase and peroxidase enzymes in the bacterial cell in strain JW3914-1 Δ*katG729::kan*, and especially in JW3933-3 Δ*oxyR749::kan*, which indicates the role of hydrogen peroxide in this process. (+)-α-Pinene in an amount of ≤5 mg (≤6 µL) almost does not inhibit the growth of *E. coli* strains (data not shown).

## 4. Discussion

It was shown that cyclic terpene limonene, when present in the growth medium during incubation for several hours, destroys the cell membrane in yeast cells (*Z. rouxii*) and bacterial cells (*E. coli* and *S. aureus*), which is accompanied by leakage of nucleic acids and proteins [8,13]. Moreover, the stereochemistry of limonene and α-pinene influences antimicrobial activity: in general, (−)-limonene and (+)-α-pinene are more active, and their antimicrobial activity is pathogen-specific [10,11,12]. Highly sensitive specific lux-biosensors were used in the present work to study the effects of (−)-limonene and (+)-α-pinene and determine the mechanism of action of the above-mentioned VOCs on bacterial cells. We found that starting from a concentration of 1 µM, (−)-limonene induces synthesis of considerable amounts of H_2_O_2_ in the bacteria within the first 20–30 min of incubationand induces the synthesis of superoxide anion radicals after 30 min. Moreover, this VOC causes damage to DNA and proteins after 40–50 min of action. We assume that the induction of oxidative stress, observed at the first stage (minutes) of the interaction of (−)-limonene with bacterial cells and associated with the synthesis of reactive oxygen species (ROS)—hydrogen peroxide and superoxide anion radical—is of considerable interest to researchers who use terpene derivatives. Previous studies have determined the reaction of cells to limonene at the late stage (hours) and recorded the damage to membranes. However, when using limonene as an inhibitor of yeast and bacteria in preparations of nutritious juices and other food products, it is necessary to know that limonene not only inhibits bacteria and yeast (i.e., it acts as an antibiotic on a specific target) but also induces the formation of ROS that can be dangerous to the human body. A similar situation arose in the study of the action of antibiotics when J. J. Collins et al. (USA) [43,44,45] found that antibiotics, aside from acting on the primary target (membranes, DNA-gyrase, etc.), induce the formation of a significant amount of ROS in the cell. This caused a great interest in the problem since ROS, penetrating human cells, cause DNA damage, mutations, and cancer degenerations.

As reported in [46], intracellular targets of 2,4-diacetylphloroglucinol action were assessed using bacterial biosensors with inducible bioluminescence corresponding to DNA and protein damage. However, unlike our study, it was not possible to register a positive response from any biosensor. As a result, the bactericidal effect of 2,4-DAPG is believed to be related to the destruction of bacterial barrier structures [46].

The data obtained in this work show that the observed effects (oxidative stress, SOS response, etc.) are unique since the investigated range of concentrations of (−)-limonene (<10 μM) weakly inhibits the enzyme-reporter activity of luciferase. Inhibition of the luciferase enzymatic activity by (−)-limonene observed at concentrations ≥10 μM (Figure 8) is reversible. It can be explained by the competition of VOC with the luciferase substrate, long-chain aldehyde, and/or FMNH_2_. This effect should be taken into account when working with luxbiosensors. In vitro, the reversible inhibition of the bacterial luciferase was determined for several compounds competitive with FMNH_2_ and long-chain aldehyde [47,48,49].

Summarizing the obtained data on the induction of oxidative stress by studied VOCs, it can be concluded that only (−)-limonene in a bacterial cell contributes to the synthesis of a significant amount of hydrogen peroxide and superoxide anion radical. On the other hand, the mechanism of the formation of reactive oxygen species (ROS) remains unclear. Apparently, for (+)-α-pinene, the mechanism of action on bacterial strains does not relate to forming ROS.

The method of using bacteria strains containing eukaryotic luciferase *Pyrophorus plagiophtalamus* (click beetle) activated by D-luciferin to measure membrane permeability was first applied in the analysis of the action of low-molecular membrane-lytic agents—cationic peptides—that form pores in biological membranes [50]. It was shown that melittin produced by *Apis mellifera* bees and the antibiotics polymyxin B and gramicidin S, at concentrations in the range of 10–100 µg/mL, completely inhibit the growth of *E. coli* MC1061 bacteria when seeding on Petri dishes. Furthermore, a significant increase in the luminescence of cells containing firefly luciferase was observed at the same concentrations, indicating an increase in the membrane permeability for D-luciferin [50]. However, unlike (−)-limonene, which gradually induces bioluminescence increase in cells within 30–50 min of incubation (Figure 10A), cationic peptides cause an increase in the luminescence of the cell suspension within a few seconds after being added to the cells, the passage of luminescence through a maximum within 20–30 s, and a relatively rapid decrease in luminescence over the next 2 min. Therefore, in the case of (−)-limonene, a much more moderate, prolonged effect of the toxic agent on the bacterial membrane structure is observed. Besides, the action of (−)-limonene does not lead to a decrease in the intracellular content of ATP compared to the mechanisms of cationic peptides and CCCP action.

The response of the luxbiosensor with the P*ibpA* promoter, which fixes the heat shock, to the action of (−)-limonene and (+)-α-pinene is of particular interest and is shownfor the first time. As can be seen from the obtained data presented in Figure 7, (+)-α-pinene induces the opening of the heat shock promoter P*ibpA* only during the longer incubation time compared to the action of (−)-limonene. However, (+)-α-pinene does not induce ROS formation in the cell (Figure 2 and Figure 3). (+)-α-Pinene, like (−)-limonene, inhibits refolding carried out by the DnaKJE chaperone, but only partially and only in the Δ*ibpB* mutant strain lacking the small chaperone IbpB (Figure 9). These results are consistent with the data on the effects of ketones 2-heptanone, 2-nonanone, and 2-undecanone that were reported by us earlier [36]. It can be assumed that (−)-limonene and (+)-α-pinene, as hydrophobic compounds, are capable of complexing with the corresponding regions of the DnaKJE, hindering the chaperone protein, and inhibiting its ability to form a complex not only with denatured proteins but also with σ32 (the subunit σ32 is responsible for the synthesis of “heat shock” promoters [51,52,53,54]).

The effect of complete inhibition of the ATP-dependent chaperone (DnaKJE) activity by (−)-limonene should also be of considerable interest. Currently, some laboratories are searching for organic molecules that can inhibit ATP-dependent chaperones and exhibit an anticancer effect [55,56,57]. Several thousands of synthesized organic compounds have been tested, and only a few of them can hinderchaperones to some extent. However, as a rule, such compounds are toxic to the human body. In this work, we found that a natural product contained in citrus fruits completely and at very low concentrationsinhibits the DnaK-dependent refolding, which can be of practical use.

## 5. Conclusions

The initial stage of the action of (−)-limonene on the cell is the induction of oxidative stress, namely the formation of a significant amount of ROS (hydrogen peroxide and superoxide anion radical), which damages DNA (SOS response). At the same time, bacterial membranes are damaged, which leads to an increase in their permeability. Moreover, heat shock is induced by direct contact of (−)-limonene and (+)-α-pinene with the DnaKJE–σ32 complex.

The action of (−)-limonene at high concentrations and prolonged incubation time makes degrading processes in cells irreversible. This leads to the destruction of the membrane and the release of nucleic acids and proteins outside, which results in the lysis of bacteria.

## Figures and Tables

**Table 1:** *Escherichia coli* strains used in this study.

Strain	CGSC ** ID	Genotype	Reference	Comments
MG1655	6300	F^-^*ilvG rfb-50 rph-1*	[22]	Prototroph
BW25113	7636	F^−^, Δ*(araD-araB)567*, Δ*lacZ4787*(::rrnB-3), *л*^−^, *rph-1*, Δ*(rhaD-rhaB)568*, *hsdR514*	[23]	(This is) The parent strain for the Keio Collection of single-gene knockouts.
JW3914-1	10827	Δ*katG729::kan*	[24]	Keio Collection strain
JW3663	10689	*ibpB::kan*	[24]	Keio Collection strain
JW3933-3	12039	Δ*oxyR749::kan*	[24]	Keio Collection strain
QC868	no	F*^−^leu6 thrA1 proA2 thi-1 lacY1 tonA1 rpsL31 supE44 hsdR^−^sodA^+^sodB^+^*Sm^r^	[25]	The parent strain for QC871
QC871	no	F*^−^leu6 thrA1 proA2 thi-1 lacY1 tonA1 rpsL31 supE44 hsdR^−^sodA25 sodB2*Cm^r^Kan^r^Sm^r^	[25]	The double mutant (*sodAB*^−^) without the manganese- and iron-cofactored superoxide dismutases

** CGSC—The Coli Genetic StockCenter.

**Table 2:** Diameters of inhibition zone (DIZs) of different *E. coli* strains afterincubation with (−)-limonene (4 µL (3.36 mg)).

*E. coli* Strain	DIZ, mm *
MG1655 (wt)	7 ± 0.5 **
QC871 *sodAB*^-^	8 ± 0.5
JW3933-3 Δ*oxyR749::kan*	16 ± 0.5
JW3914-1 Δ*katG729::kan*	10 ± 0.5

* All the assays were carried out in triplicate. The data were recorded as mean ± standard error The same results were obtained for BW25113 and QC868.

## Data Availability

The author elects not to share data.
